# Reduction in Non-Protein Respiratory Quotient Is Related to Overall Survival after Hepatocellular Carcinoma Treatment

**DOI:** 10.1371/journal.pone.0055441

**Published:** 2013-03-08

**Authors:** Masaya Saito, Yasushi Seo, Yoshihiko Yano, Kenji Momose, Hirotaka Hirano, Masaru Yoshida, Takeshi Azuma

**Affiliations:** 1 Division of Gastroenterology, Department of Internal Medicine, Kobe University Graduate School of Medicine, Kobe, Japan; 2 Center for Infectious Diseases, Kobe University Graduate School of Medicine, Kobe, Japan; 3 Division of Metabolomics Research, Department of Internal Medicine, Kobe University Graduate School of Medicine, Kobe, Japan; University of Modena & Reggio Emilia, Italy

## Abstract

**Background:**

Transcatheter arterial chemoembolization (TACE) is an effective treatment for hepatocellular carcinoma (HCC) that can occasionally lead to the shortening of life expectancy. We aimed to make a new and more accurate prognostic model taking into account the course of disease after TACE.

**Methodology/Principal Findings:**

We performed a prospective cohort study involving 100 HCC patients who underwent TACE at Kobe University Hospital. Indirect calorimetry and blood biochemical examinations were performed before and 7 days after TACE. Time-dependent and time-fixed factors associated with 1-year mortality after TACE were assessed by multivariate analyses. A predictive model of 1-year mortality was established by the combination of odds ratios of these factors. Multivariate analyses showed that the ratio of non-protein respiratory quotient (npRQ) (7 days after/before TACE) and Cancer of Liver Italian Program (CLIP) score were independent factors of 1-year mortality after TACE (p = 0.014 and 0.013, respectively). Patient-specific 1-year mortality risk scores can be calculated by summarizing the individual risk scores and looking up the patient-specific risk on the graph.

**Conclusions:**

The short-term reduction of npRQ was a time-dependent prognostic factor associated with overall survival in HCC patients undergoing TACE. CLIP score was a time-fixed prognostic factor associated with overall survival. Using the prediction model, which consists of the combination of time-dependent (npRQ ratio) and time-fixed (CLIP score) prognostic factors, 1-year mortality risk after TACE would be better estimated by taking into account changes during the course of disease.

## Introduction

Transcatheter arterial chemoembolization (TACE) is the most widely used first-line treatment in the Western world and Asia for patients with unresectable hepatocellular carcinoma (HCC). TACE displays a tumor-response rate of 30–60% [1]–[3] and shows a survival benefit compared with control [4]–[6]. However, TACE against HCC sometimes worsens liver parenchymal function. A deterioration of liver function due to ischemia of the non-tumoral liver following TACE can occasionally lead to the shortening of life expectancy [7]–[9]. Current guidelines for HCC management recommend mortality risk estimates as a decision-making support [10].

Several staging systems before HCC treatment have been developed for mortality risk estimates. The best known systems are as follows: Child’s score [11], Model for End-stage Liver Disease (MELD) score [12], Tumor Node Metastasis (TNM) stage [13], Barcelona Clinic Liver Cancer (BCLC) stage [14], Cancer of Liver Italian Program (CLIP) score [15], [16], Japanese Integrated System (JIS) score [17], [18], and Okuda score [19]. However, all previous prognostic factors can only predict survival at a single time point for each patient, but not predict it from the standpoint of the clinical and biological alterations in HCC patients after treatment. Key issues for prognostic prediction in HCC patients are to establish new and more accurate time-dependent prognostic factors taking into account the alterations during the course of disease [20]. Recently, Cabibbo et al. showed that performance status, prior therapy, number of treatments, complete response after TACE, and bilirubin level were associated with overall survival using a time-dependent model [21]. On the other hand, our recent studies demonstrated that the short-term alteration of energy metabolism could be associated with the long-term deterioration of liver function in HCC patients after TACE [22]. We also showed that the long-term deterioration of liver function could be related to overall survival in HCC patients after TACE [23]. In this study, long-term follow-up of the HCC patients after TACE was performed. To our knowledge, there have been no studies showing that biological alterations after HCC treatment, such as dynamics of energy metabolism, could contribute to prediction of overall survival.

The aims of this prospective cohort study of HCC patients undergoing TACE were (i) to establish new time-dependent biological predictive factors of overall survival; (ii) to compare the time-dependent new biological predictive factors with the time-fixed previous prognostic factors in terms of the predictive ability of overall survival; and (iii) to create a score card by rounding off the odds ratios of both time-dependent and time-fixed factors in the multivariate model.

## Patients and Methods

### Ethics Statement

All patients provided written consent to participate in the study, and the protocol was approved by the institutional ethics review board at Kobe University. This study complies with the standards of the Declaration of Helsinki and current ethical guidelines. The URL of the online registry is www.umin.ac.jp/ctr/index.htm, and the clinical trial number is UMIN000004273.

### Patients

We performed a prospective cohort study on a total of 100 patients (mean age: 70.9 years, range: 41–87 years; male:female ratio: 61∶39) who underwent TACE for HCC between December 2008 and April 2010 at Kobe University Hospital. All patients were Japanese and had liver cirrhosis that had been diagnosed on the basis of laboratory data and ultrasonography. According to the modified Child’s classification [11], 58 patients were classified into Child’s grade A and 42 were classified into grade B. The etiology of cirrhosis was as follows: hepatitis B in 12 patients, hepatitis C in 71, alcoholic liver dysfunction in 28, primary biliary cirrhosis in 2, autoimmune hepatitis in 2, non-alcoholic fatty liver disease in 5, and unknown in 1, although each category overlaps with others. The diagnosis of HCC was based on findings obtained from contrast-enhanced X-ray computed tomography (CT) scans and hepatic-artery angiography. Ascites was confirmed by ultrasonography and CT.

In Japan, a consensus-based clinical practice manual proposed by the Japan Society of Hepatology is widely used for the management of HCC [24]. Those who had 4 or more lesions, those who had 3 or fewer lesions and tumor of 3 cm or more in size, and those who had mild portal invasion without an extra-hepatic lesion were eligible for this study. In addition, those who did not consent to a liver resection, those who were difficult cases in terms of undertaking radiofrequency ablation (RFA) and percutaneous ethanol injection (PEI), and those who were elderly, that is, over 80 years old, were also eligible. Those who had refractory ascites, overt encephalopathy, uncontrolled esophageal varices, severe jaundice, Child’s grade C, or portal vein trunk thrombosis were excluded from the study. In addition, those who used intravenous albumin regularly, those who were given a branched-chain amino acids (BCAA) preparation, and those who had dialysis were also excluded. Liver function of these cases was limited to Child’s grade A or B.

A follow-up time was defined as the number of months from TACE to last contact with the patient or death. All patients were periodically seen as outpatients at our hospital. The follow-up protocol included clinical assessment by physical examination and biochemistry every month and the use of ultrasonography or contrast-enhanced CT scan every 3 months. In our study, TACE-related mortality was defined as death from a complication within 2 weeks of each session of TACE.

### Clinical evaluation

The clinical evaluation before treatment was performed on the basis of results of physical examination and laboratory measurements. All patients were analyzed by the Child’s score, MELD score, TNM stage, BCLC stage, CLIP score, JIS score, modified-JIS score, and Okuda score. The total kcal/day oral intake per day was monitored 7 days before and after TACE. The total oral intake before TACE was averaged, from the day on which it was performed and 7 days later. The calculation of the total oral intake of each patient was based on the following conditions: total calories for ideal body weight (IBW) (a body mass index (BMI) of 22): 30–35 kcal/kg per day, 1–1.5 g protein/kg per day, fat content equivalent to 25% of total calories. The duration (days) of febrile episodes (over 37°C) after TACE was recorded, and the therapeutic volume (cm^3^) in TACE was evaluated by observing Lipiodol deposits on a liver CT scan following TACE. Past TACE treatment frequency (number of times) and other treatments (surgical resection, RFA, and PEI) were also documented. Laboratory measurements including lymphocytes, aspartate aminotransferase (AST), alanine aminotransferase (ALT), γ-glutamyltranspeptidase (γ-GTP), total bilirubin (T-Bil), albumin (Alb), prealbumin (preAlb), cholinesterase (ChE), C-reactive protein (CRP), the branched-chain amino acid/tyrosine ratio (BTR), prothrombin time (PT), and PT-international normalized ratio (INR) were assessed before and 7 days after TACE. Platelets (Plt), creatinine (Crn), fasting plasma glucose (FPG), immunoreactive insulin (IRI), hemoglobin A1c (HbA1c), type 4 collagen 7S, hyaluronic acid (HA), alpha fetoprotein (AFP), des-γ-carboxy prothrombin (DCP), and the indocyanine green dye clearance test (ICG test) retention rate at 15 min were only examined before TACE.

### Protocol for TACE

TACE for HCC was performed by catheterization via the femoral artery under local anesthesia, with super-selective catheterization of the hepatic artery feeding the tumor, unless bilobar tumors were involved, in which case chemoembolization was performed in the appropriate hepatic artery. Depending on the tumor size, various amounts of an emulsion of 20 mg of Farmorubicin (epirubicin hydrochloride; Pfizer, USA) and 3 ml of Lipiodol (the iodine addition products of the ethyl esters of fatty acids obtained from poppy seed oil; Mitsui, Japan) in a 1∶1 volume ratio were injected under fluoroscopic monitoring. This was followed by embolization with gelatin (Gelpart; Yamanouchi, Japan). Following TACE, a liver CT scan was performed to calculate the therapeutic volume from the distribution of Lipiodol deposits.

### Energy metabolism

Energy metabolism was analyzed using indirect calorimetry (Deltatrac II Metabolic Monitor from Datex Division Inst. Corp., Helsinki, Finland). Indirect calorimetry was performed for 30 minutes after overnight bed rest and fasting. The time that has elapsed from that meal until indirect calorimetry is performed was constant (about 11 hours) in all of the patients. The patients were kept in a supine position in bed and allowed to breathe ambient air during the examination. Oxygen consumption per minute, carbon dioxide production per minute, non-protein respiratory quotient (npRQ), and resting energy expenditure (REE) were measured before and 7 days after TACE.

### Statistical analysis

Statistical analyses were conducted using SPSS Statistics 18.0 (SPSS Inc., Chicago, IL, USA). Cochran-Armitage test was performed to identify the linearity of each factor. Variance inflation factor (VIF) was used to identify multicollinearity of the factors. A multivariate model was constructed using forward selection logistic regression analyses. Akaike’s Information Criterion (AIC) was calculated to confirm the optimal number of factors to include in the predictive model. The Hosmer Lemeshow statistic for goodness of fit was calculated to evaluate the fitness of the multivariate logistic model. Likelihood ratio (LR) was used to evaluate the predictive performance of each significant multivariate factor. The predictive ability of the model was quantified using each significant multivariate factor and 1-year mortality (area under ROC, AUC). The DeLong method with Bonferroni correction was performed to make pairwise comparisons of ROC curves. The optimal cut-off point was the score that produced the maximal sum of sensitivity and specificity. Log-rank test was performed to compare the survival distributions. Values of P<0.05 were considered significant.

A clinical risk score card was created to assist clinicians in identifying patients at risk of 1-year mortality after TACE. Odds ratios from the multivariate model represented the score for that risk factor. Patients were stratified by score and the rate was calculated using the observed (1-year mortality) result for each score. This event rate was then plotted and a fitted trend line was estimated across the categories.

## Results

### Factor analyses of 1-year mortality after TACE

One-year mortality after TACE was found in 22 patients out of 100 (1-year mortality group), and 1-year survival was detected in 78 patients (1-year survival group). Table 1 shows the results of univariate analyses comparing several factors before TACE between the two groups. Height, BMI, Child’s score, TNM stage, BCLC stage, CLIP score, JIS score, modified-JIS score, Okuda score, past treatment frequency of TACE, and DCP were significantly higher in the 1-year mortality group than in the survival group (p = 0.023, 0.012, 0.038, 0.0004, 0.0000, 0.0000, 0.0001, 0.0002, 0.001, 0.0005, and 0.0002, respectively). The total oral intake (kcal/IBW/day) showed no significant differences between the two groups (p = 0.080), and was not also correlated with npRQ (p = 0.646).

**Table 1:** The background clinical characteristics of the one-year mortality group and the survival group after TACE (n = 100).

Factor	One-year mortality group	One-year survival group	p value
	n = 22	n = 78	
Age (years)	74 (56–86)	71 (41–87)	0.436†
Sex (male/female)	12/10	49/29	0.621¶
Height (cm)	156.1±8.2 (144.0–170.7)	160.7±9.6 (138.0–179.0)	0.023*
Body weight (kg)	59.5±7.9 (47.8–74.7)	59.3±10.8 (37.0–93.6)	0.468*
BMI (kg/m^2^)	24.4±2.6 (19.0–28.8)	22.8±2.9 (15.6–30.2)	0.012*
HBs Ag (+/−)	3/19	9/69	0.723¶
HCV Ab (+/−)	17/5	54/24	0.598¶
Alcohol (over 20 g/day)(+/−)	4/18	24/54	0.293¶
Child's score (5, 6, 7, 8, 9)	3/7/5/4/3	35/13/12/11/7	0.038†
MELD score	7.4 (6.0–11.9)	7.1 (6.0–20.7)	0.403†
TNM Stage of HCC (I, II, III, IVa, IVb)	0/5/13/4/0	9/32/36/1/0	0.0004*
BCLC Stage of HCC (0, A, B, C, D)	0/0/19/3/0	9/34/34/1/0	0.0000*
CLIP score (0, 1, 2, 3, 4, 5, 6)	1/2/7/8/4/0/0	21/24/28/5/0/0/0	0.0000*
JIS score (0, 1, 2, 3, 4, 5)	0/1/10/10/1/0	7/19/38/14/0/0	0.0001*
Modified-JIS score (0, 1, 2, 3, 4, 5)	0/1/10/8/3/0	7/18/37/15/1/0	0.0002*
Okuda score (I, II, III)	3/19/0	44/34/0	0.001¶
Past treatment frequency of TACE (times)	3 (0–11)	1 (0–10)	0.0005†
Past treatments other than TACE (+/−)	12/10	26/52	0.085¶
Total oral intake (kcal/IBW/day)	32.3±4.4 (27.0–43.8)	30.9±4.5 (18.9–42.7)	0.080*
npRQ	0.88 (0.74–1.16)	0.87 (0.71–1.31)	0.112†
REE (kcal)	1284±177 (930–1560)	1348±229 (840–2030)	0.115*
Plt (×10^4^/µl)	9.3 (4.0–23.7)	10.2 (1.1–23.1)	0.163†
Lymphocytes (/µl)	1094 (519–1879)	1094 (299–3143)	0.868†
PT (%)	77.1 (67.2–99.5)	84.2 (57.6–100.0)	0.188†
AST (IU/l)	46 (19–127)	50 (18–162)	0.188†
ALT (IU/l)	35 (11–89)	38 (13–131)	0.148†
γ-GTP (IU/l)	41 (10–310)	50 (13–1188)	0.209†
T-Bil (mg/dl)	0.7 (0.4–2.5)	0.9 (0.3–2.4)	0.162†
ChE (IU/l)	149±65 (81–348)	174±71 (47–327)	0.071*
Alb (g/dl)	3.2 (2.4–4.3)	3.7 (2.4–4.8)	0.060†
PreAlb (mg/dl)	9.6 (4.5–19.0)	13.0 (4.2–33.1)	0.055†
BTR	3.7±1.4 (1.7–7.4)	4.1±1.5 (1.5–8.0)	0.160*
CRP	0.1 (0.1–3.5)	0.1 (0.1–6.9)	0.501†
Crn	0.72 (0.46–1.13)	0.76 (0.44–4.91)	0.644†
FPG (mg/dl)	102 (88–207)	101 (74–351)	0.360†
IRI (µU/ml)	14 (2–40)	11 (1–84)	0.165†
HOMA-IR	3.48 (0.62–12.10)	2.58 (0.19–43.30)	0.166†
HbA1c (%)	5.4 (4.5–7.7)	5.3 (4.1–10.2)	0.937†
ICG test retention rate at 15 min (%)	28.9 (8.5–75.6)	22.1 (3.5–68.0)	0.182†
Type 4 collagen 7S (ng/ml)	7.2 (4.2–10.0)	6.6 (3.0–16.0)	0.364†
HA (ng/ml)	297.8 (88.5–1160.3)	299.6 (12.4–3968.6)	0.840†
AFP (ng/ml)	80 (5–94260)	17 (2–14183)	0.058†
DCP	453 (16–148950)	91 (14–7138)	0.0002†

Data represent n, mean±SD (range), or median (range).

The data were evaluated with the *two-sample t-test, †Wilcoxon rank sum test, or ¶Fisher's exact test as appropriate.

Table 2 shows the results of univariate analyses comparing the ratios of several factors (7 days after/before TACE) between the two groups. npRQ, PT, ChE, Alb, and preAlb in the 1-year mortality group were significantly lower than those in the survival group (p = 0.0003, 0.039, 0.0001, 0.006, and 0.001, respectively). T-Bil and ALT in the 1-year mortality group were significantly higher than those in the survival group (p = 0.002 and 0.042, respectively). Therapeutic volume by TACE was significantly higher in the 1-year mortality group than in the survival group (p = 0.003). CRP, total oral intake, and febrile duration (days) showed no significant differences between the two groups (p = 0.054, 0.092, and 0.257, respectively), and were not also related to npRQ (p = 0.052, 0.167, and 0.135, respectively)

**Table 2:** Univariate analyses comparing several factor ratios (7 days after/before TACE) between the one-year mortality group and the survival group after TACE (n = 100).

Factor ratio	One-year mortality group	One-year survival group	p value
(7 days after/before TACE)	n = 22	n = 78	
npRQ	0.95 (0.76–1.04)	1.00 (0.81–1.25)	0.0003†
REE	1.01 (0.88–1.12)	0.97 (0.78–1.22)	0.125†
Lymphocytes	0.70 (0.40–2.23)	0.81 (0.41–2.22)	0.112†
PT	0.92 (0.60–1.11)	0.96 (0.79–1.23)	0.039†
T-Bil	1.53 (0.80–2.26)	1.16 (0.50–3.00)	0.002†
ChE	0.70±0.10 (0.55–0.91)	0.83±0.15 (0.47–1.17)	0.0001*
Alb	0.83±0.06 (0.67–0.96)	0.89±0.10 (0.59–1.17)	0.006*
PreAlb	0.52 (0.34–0.78)	0.66 (0.22–1.56)	0.001†
AST	1.03 (0.54–2.13)	0.89 (0.30–3.92)	0.068†
ALT	1.65 (0.84–17.67)	1.30 (0.36–12.08)	0.042†
γ-GTP	1.05 (0.79–3.11)	0.97 (0.52–1.86)	0.306†
BTR	1.13±0.33 (0.52–1.71)	1.10±0.27 (0.63–1.94)	0.694*
CRP	16.5 (1.3–97.2)	8.2 (0.1–62.7)	0.054†
Total oral intake	0.8 (0.2–1.0)	0.9 (0.1–1.0)	0.092†
Febrile duration (days)	4 (0–7)	3 (0–7)	0.257†

Data represent mean ± SD (range) or median (range).

The data were evaluated with the *two-sample t-test or †Wilcoxon rank sum test as appropriate.

P values shown in bold are statistically significant.

JIS score consists of Child’s score and TNM stage, so JIS score, Child’s score, and TNM stage were clinically considered to have multicollinearity with each other. Child’s score and TNM stage were not introduced into the multivariate logistic regression analyses. Height and BMI were also clinically considered to have multicollinearity, so height was not introduced into the multivariate analyses. The 16 significant univariate factors (BMI, BCLC stage, CLIP score, JIS score, modified-JIS score, Okuda score, past treatment frequency of TACE, therapeutic volume by TACE, DCP, npRQ ratio, PT ratio, T-Bil ratio, ChE ratio, Alb ratio, preAlb ratio, and ALT ratio) displayed linearity in the Cochran-Armitage test, and these factors were used as continuous variables. VIF was less than 5 among all of the 16 significant univariate factors. No multicollinearity was detected among the 16 significant univariate factors, so all of them were introduced into the multivariable logistic model.

The forward selection multivariate logistic regression analyses consisted of three steps (Table 3). In the final step of the multivariate model, the AIC was 60.637, the minimum value obtainable in three steps. The optimal number of factors was three (the npRQ ratio, BCLC stage, and CLIP score). The Hosmer Lemeshow test showed the fitness of the final step of the multivariate model (χ^2^ = 0.984, p = 0.998). The npRQ ratio, BCLC stage, and CLIP score were independent factors associated with 1-year mortality after TACE (p = 0.014, 0.010, and 0.013, respectively).

**Table 3:** Forward selection logistic regression analyses comparing the significant univariate factors in Tables 1 and 2.

Step	Factor	B	SE	p value	Exp(B)	95% CI of EXP(B)	
						upper limit	lower limit
Step 1	CLIP score	1.40	0.354	0.000	4.070	2.035	8.140
	constant	−3.93	0.807	0.000	0.020		
Step 2	BCLC stage	2.65	1.044	0.011	14.142	1.826	109.508
	CLIP score	0.90	0.364	0.014	2.458	1.204	5.020
	constant	−7.78	2.088	0.000	0.000		
Step 3	npRQ ratio	−13.87	5.654	0.014	9.48E-07	1.46E-11	0.062
	BCLC stage	2.77	1.080	0.010	15.963	1.923	132.499
	CLIP score	0.93	0.376	0.013	2.533	1.212	5.295
	constant	5.52	5.427	0.309	249.744		

B: regression coefficient; CI: confidence interval; Exp(B): odds ratio; SE: standard error.

### Prediction for 1-year mortality after TACE

**Figure 1:**
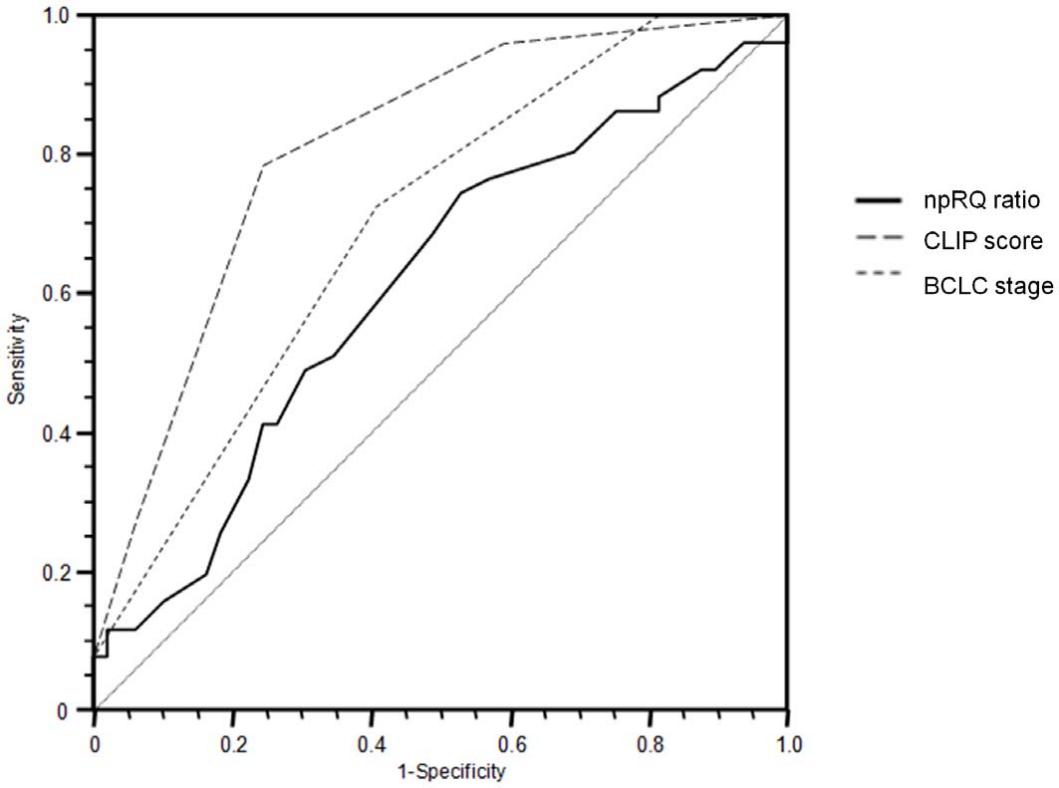
Pairwise comparison of ROC curves of time-dependent and time-fixed prognostic factors associated with 1-year mortality after TACE. Using the DeLong method with Bonferroni correction, pairwise comparison of ROC curves was performed. There were significant differences between the AUC of npRQ ratio and that of CLIP score (p = 0.019), and no significant differences between the AUC of npRQ ratio and that of BCLC stage (p = 0.592). There were no significant differences between the AUC of BCLC stage and that of CLIP score (p = 0.094), but The AUC of CLIP score was higher than that of BCLC stage (0.811 vs. 0.700). So, CLIP score was a better index than the BCLC stage.

The values of LR of npRQ ratio, BCLC stage, and CLIP score were 10.256, 31.713, and 30.792, so each factor displayed a significantly good performance for the prediction of 1-year mortality after TACE (p = 0.001, 0.000, and 0.000, respectively). Moreover, each of npRQ ratio, BCLC stage, and CLIP score discriminated well between patients with and without 1-year mortality (AUC = 0.611, 0.700, and 0.811, respectively) (Table 4). Using the DeLong method with Bonferroni correction, pairwise comparison of ROC curves was performed. There were significant differences between the AUC of npRQ ratio and that of CLIP score (p = 0.019), and no significant differences between the AUC of npRQ ratio and that of BCLC stage (p = 0.592). There were no significant differences between the AUC of BCLC stage and that of CLIP score (p = 0.094) (Figure 1), but The AUC of CLIP score was higher than that of BCLC stage (0.811 vs. 0.700). In addition, CLIP score was significantly related to both TNM stage of HCC and Child’s score before TACE (p = 0.000 and 0.000, respectively), on the other hand, BCLC stage was only related to TNM stage, not to Child’s score before TACE (p = 0.000 and 0.898, respectively). So, CLIP score was a better index than the BCLC stage, and then the CLIP score was selected and the BCLC stage was dropped out. The npRQ ratio was not related to both TNM stage and Child’s score before TACE (p = 0.479 and 0.460, respectively), so the npRQ ratio was not affected by BCLC stage and CLIP score, and then the npRQ ratio was also selected.

**Table 4:** Area under ROC (AUC) and 95% confidence interval (CI) of each independent factor associated with 1-year mortality after TACE.

Multivariate factor	AUC	95% CI
npRQ ratio	0.611	0.508 to 0.707
BCLC stage	0.700	0.600 to 0.787
CLIP score	0.811	0.720 to 0.882

Each AUC of npRQ ratio, BCLC stage, and CLIP score had a good ability to discriminate between the 1-year mortality group and the survival group after TACE.

**Figure 2:**
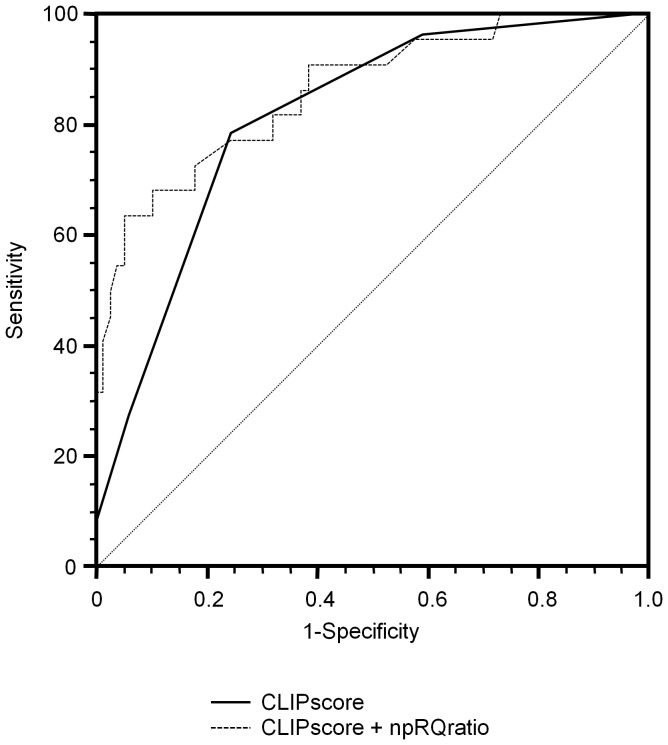
Pairwise comparison of ROC curves of CLIP score alone and combination factor (CLIP score and npRQ ratio) associated with 1-year mortality after TACE. Using the DeLong method with Bonferroni correction, pairwise comparison of ROC curves was performed. There were no significant differences between the AUC of CLIP score alone and that of combination factor (p = 0.061), but the AUC of combination factor was higher than that of CLIP score alone (0.861 vs. 0.811). The combination factor (CLIP score and npRQ score) was the best index for prediction of 1-year mortality after TACE.

Predictive score by combination of selected two factors (CLIP score and npRQ ratio) was estimated as follows: 1-(1/(1+EXP(1.451×CLIPscore-12.225×npRQ ratio+7.993))). The value of LR of the combination factor was 33.883, so the combination factor displayed a significantly good performance for the prediction of 1-year mortality after TACE (p = 0.000). The combination factor discriminated well between patients with and without 1-year mortality (AUC = 0.861, 95% confidence interval (CI): 0.778–0.922). There were no significant differences between the AUC of CLIP score alone and that of combination factor (p = 0.426), but the AUC of combination factor was higher than that of CLIP score alone (0.861 vs. 0.811). The combination factor (CLIP score and npRQ score) was the best index for prediction of 1-year mortality after TACE (Figure 2).

**Figure 3:**
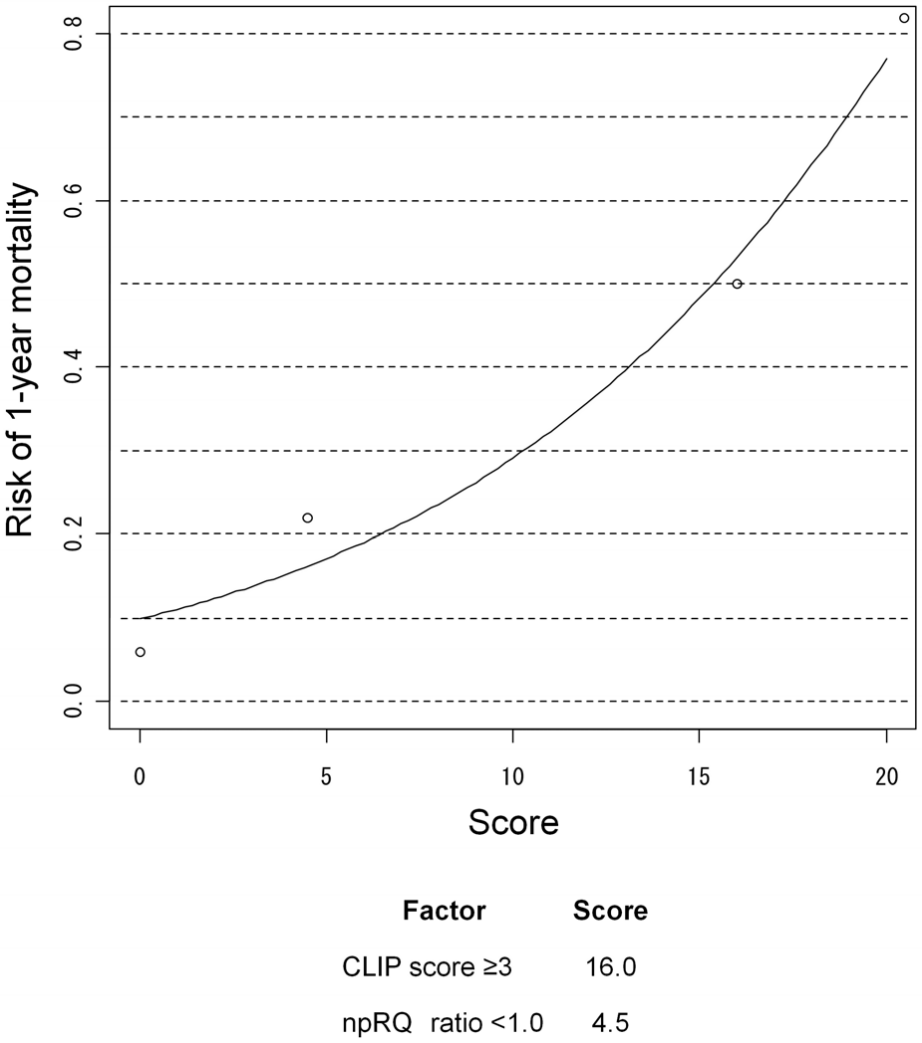
A score card implemented by rounding off the odds ratios of time-dependent and time-fixed prognostic factors associated with 1-year mortality after TACE in the multivariate model. Using the optimal cut-off points, each of npRQ ratio and CLIP score was classified into two categories. We created a score card by rounding off the odds ratios of the two factors in the multivariate model. Patient-specific 1-year mortality risk scores can be calculated by summarizing the individual risk scores and looking up the patient-specific risk on the graph.

Each optimal cut-off point of the npRQ ratio alone, CLIP score alone, and combination of CLIP score and npRQ ratio was 1.000, 3, and 0.395, respectively. The sensitivity and specificity of them were fairly good (Table 5). Using the optimal cut-off points, each of npRQ ratio and CLIP score was classified into two categories. We created a score card by rounding off the odds ratios of the two factors in the multivariate model. Patient-specific 1-year mortality risk scores can be calculated by summarizing the individual risk scores and looking up the patient-specific risk on the graph (Figure 3).

**Table 5:** Sensitivity and specificity of each independent factor for predicting 1-year mortality after TACE.

A. npRQ ratio		
Optimal cut-off point	1-year mortality group	1-year survival group
npRQ: 1.00	n = 22	n = 78
<1.00	16 (72.7%)*	27 (34.6%)
≥1.00	6 (27.3%)	51 (65.4%)**

B. CLIP score		
Optimal cut-off point	1-year mortality group	1-year survival group
CLIP score: 3	n = 22	n = 78
≤2	10 (45.5%)	73 (93.6%)**
≥3	12 (54.5%)*	5 (6.4%)

C. The combination of CLIP score and npRQ ratio		
Optimal cut-off point	1-year mortality group	1-year survival group
combination score: 0.395	n = 22	n = 78
≤0.395	8 (36.4%)	73 (93.6%)**
>0.395	14 (63.6%)*	5 (6.4%)

Predictive score by combination of CLIP score and npRQ ratio was estimated as follows: 1-(1/(1+EXP(1.451×CLIPscore-12.225×npRQ ratio+7.993))).

* Sensitivity, **Specificity.

### Follow-up

**Figure 4:**
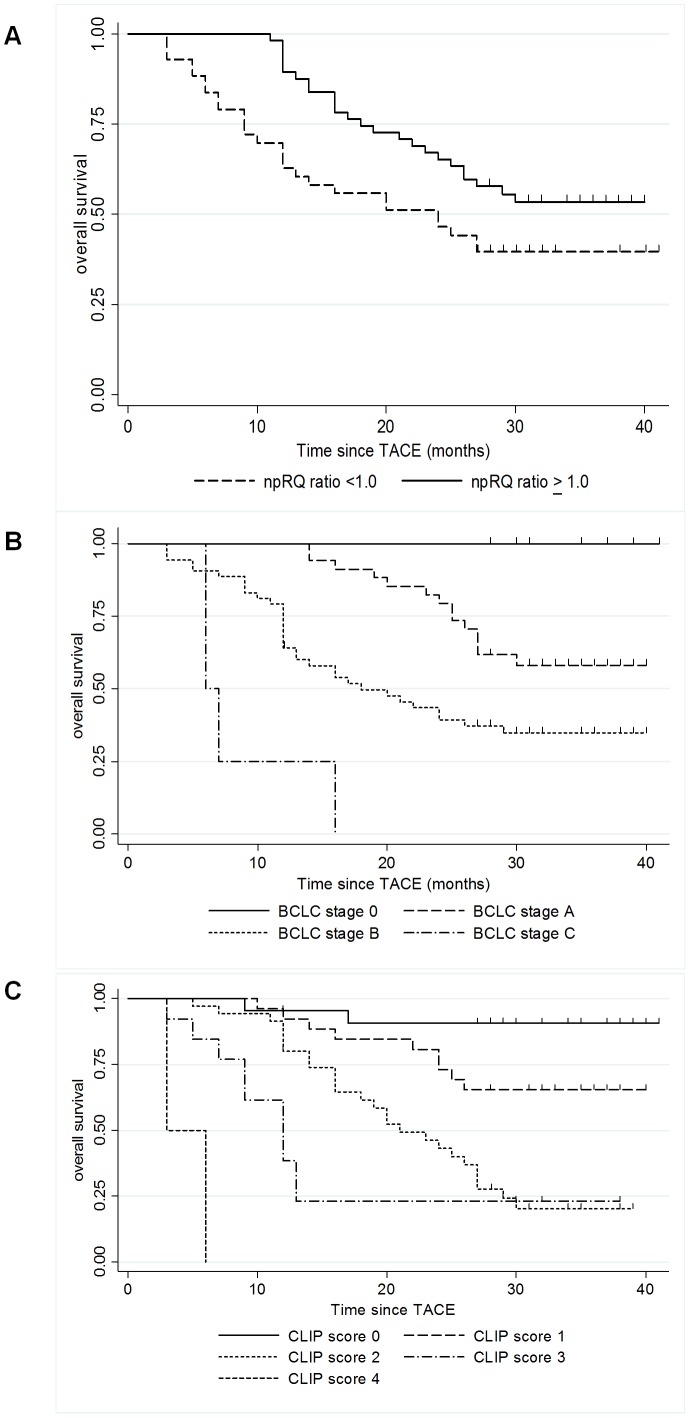
Overall survival according to each time-dependent and time-fixed prognostic factor in 100 patients with HCC after TACE. Overall survival was significantly shorter in the npRQ reduction group (npRQ ratio<1.0) than in the no-reduction group (npRQ ratio≥1.0) (p = 0.034) (Figure 3A). In addition, the higher the BCLC stage and CLIP score became, the significantly shorter the overall survival was (p = 0.000 and 0.000, respectively) (Figure 3B, C). All of these factors correctly differentiated survival for patients in different classes.

All of the 100 patients were completely followed up. During follow-up, 51 patients died, whereas 49 patients were alive at the end of the study. TACE-related mortality was not observed in this study. The median follow-up period was 27 months (3-41 months). The median survival time was 27.0 months (95% CI 25.019-30.460). The overall survival rates were 78.0% and 56.7% at 12 and 24 months, respectively. Using the Kaplan-Meier method, the distribution of the 100 patients among the different classes for each independent factor (npRQ ratio, BCLC stage, and CLIP score) associated with 1-year mortality after TACE is described in Figure 4. Overall survival was significantly shorter in the npRQ reduction group (npRQ ratio<1.0) than in the no-reduction group (npRQ ratio≥1.0) (p = 0.034) (Figure 4A). In addition, the higher BCLC stage and CLIP score became, the significantly shorter the overall survival was (p = 0.000 and 0.000, respectively) (Figure 4B, C). All of these factors correctly differentiated survival for patients in different classes.

## Discussion

Energy malnutrition is a common finding in cirrhotic patients [25], [26]. Owen et al. reported that patients with cirrhosis showed a marked reduction in glucose oxidation after overnight fasting together with enhanced fat catabolism similar to that observed in healthy controls after 2–3 days of starvation [27]. Most patients with HCC have underlying cirrhosis, which is frequently associated with energy malnutrition. A recent study demonstrated that patients with advanced HCC remained under conditions of severe energy malnutrition, even when their liver capacity was good [28]. Energy malnutrition can impair liver regeneration [29], and the strong deterioration of energy malnutrition by TACE can inhibit the recovery of liver function after TACE.

The npRQ is the main factor used to evaluate energy metabolism based on indirect calorimetry. With respect to the energy metabolism of cirrhotic patients, npRQ generally decreases in relation to the severity of liver cirrhosis [25]. TACE against HCC on a background of liver cirrhosis induces ischemic change of the non-tumoral liver, and sometimes worsens liver parenchymal function: the supply of glucose from the liver may be disturbed, and energy utilization from lipids can be accelerated. Therefore, TACE induces npRQ reduction in some HCC patients. Our recent studies showed that the npRQ reduction 7 days after TACE could predict patient-specific risk of worsening of liver function 3 months after TACE [22]. In addition, we showed that a larger decrease of npRQ 7 days after TACE was significantly related to much more deterioration of liver function 3 months after TACE [22]. More than 3 months after TACE, no patients displayed better liver function [22]. Moreover, the deterioration of liver function 3 months after TACE was related to overall survival in HCC patients [23]. The present study showed that the npRQ reduction 7 days after TACE was related to overall survival after TACE. It was considered that HCC patients with short-term reduction of npRQ after TACE would develop the deterioration of liver function through progression of energy malnutrition. The energy malnutrition is associated with high morbidity and mortality due to an increased risk of life-threatening complications [25]. Therefore, the npRQ reduction would be a significant factor in establishing overall survival of the patients.

The prognosis of HCC patients strongly depends not only on the grade of cancer spread, but also on the grade of residual liver function. CLIP score accounts for both tumor characteristics and liver function relevant to prognostic assessment for patients with HCC. As described in previous studies [30], [31], the present study showed that CLIP score before TACE was an independent factors associated with overall survival after TACE. The present study showed that the predictive ability of 1-year mortality by CLIP score was better than that by npRQ ratio. In terms of the reason for this, it was thought that CLIP score correlated with both grade of liver function and cancer spread before TACE, whereas npRQ ratio did not correlate with the both grade of liver function and cancer spread before TACE because liver function before TACE would not affect protein-energy malnutrition after TACE, and cancer cells would not play any supportive roles of protein-energy malnutrition. However, CLIP score were time-fixed prognostic factors before TACE and it was not capable of predicting survival of patients by taking into account changes during the course of disease and the interaction between treatment and deterioration of liver function. The npRQ ratio would reflect interaction between treatment and deterioration of liver function. Many hepatologists have truly needed new and more accurate prognostic models that include important biological factors and models that account for changes during the course of the disease [20]. We considered that the prognosis of patients after TACE would be better estimated by the combination of time-dependent (npRQ ratio) and time-fixed (CLIP score) prognostic factors. A prediction card (Figure 3) was developed and could be used to risk-stratify patients in terms of their potential for 1-year mortality.

Using the prediction card, patients who have a potentially high risk of 1-year mortality after TACE could be detected. The card is not available for the selection of HCC patients for whom TACE would not be recommended. For early detection and prevention of 1-year mortality risk after TACE, the prediction card is available. To prevent 1-year mortality risk after TACE, nutritional support is thought to be necessary in cirrhotic patients with advanced HCC undergoing TACE. Previous studies have shown that the oral BCAA supplementation improved npRQ in cirrhotic patients with or without HCC [28], [32], [33]. In addition, nutritional supplementation with oral BCAA was beneficial in reducing the morbidity rate and the rate of development of ascites, and in improving the liver function and quality of life in HCC patients undergoing TACE [29]. Our predictive model was established by the pure alteration of the npRQ after TACE without a secondary effect by BCAA supplementation. Therefore, using the predictive card, appropriate nutritional support could be administered to HCC patients undergoing TACE while avoiding the provision of unnecessary nutritional support to patients with a low risk of 1-year mortality after TACE.

When using the predictive card, there was a problem that measurement of npRQ was limited in daily practice because of the high cost of indirect calorimetry. However, a recent study showed that %arm circumference and regression equation-based npRQ could represent calorimetry-measured npRQ as parameters of energy nutrition in liver cirrhosis [34]. The alternative marker of npRQ could be useful in a routine clinical setting, and the prediction card would be available worldwide.

In conclusion, our study showed that the short-term reduction of npRQ was a time-dependent prognostic factor associated with overall survival in HCC patients undergoing TACE. Our study also showed that CLIP score was a time-fixed prognostic factors associated with overall survival. Using the prediction card, which consists of the combination of time-dependent (npRQ ratio) and time-fixed (CLIP score) prognostic factors, 1-year mortality risk after TACE would be better estimated by taking into account changes during the course of disease. It was thought that the predictive ability by combination of CLIP score and npRQ ratio was not sufficient because the number of patients in 1-year mortality group was small. Therefore, large-scale studies should be conducted to clarify new time-dependent prognostic factors, and to improve further the ability of our model to predict overall survival after TACE. In addition, we have not yet validated the predictive model. At present, we are recruiting a different cohort to validate its ability, which we will report on in the near future.
